# Supplementary material for: Mass Spectrometry Characterization of the SDS-PAGE Protein Profile of Legumins and Vicilins from Chickpea Seed
Source: Foods. 2024 Mar 14;13(6):887. doi: 10.3390/foods13060887 (PMC10969193; doi:10.3390/foods13060887)
Supplement: Supplementary file 1 [file foods-13-00887-s001.zip › Supplem Material - SDS-PAGE MS Chickpea - Foods MDPI.pdf]

foods MDPI

Supplementary Material

An in-depth Mass Spectrometry Characterization of the SDS-PAGE Protein Profile of Legumins and Vicilins from Chickpea Seed

Antonella Di Francesco [1], Michele De Santis [2], Aldo Lanzoni [1], Maria Giovanna Gaetana Pittalà [1], Rosaria Saletti [1], Zina Flagella [2], and Vincenzo Cunsolo [1,*]

[1] Laboratory of Organic Mass Spectrometry, Department of Chemical Sciences, University of Catania, Viale A. Doria 6-I, 95125, Catania, Italy
[2] Department of the Sciences of Agriculture, Food and Environment, University of Foggia, Via Napoli 25, 71122, Foggia, Italy
* Correspondence: vcunsolo@unict.it

Figures S1-S13. Graphical representation of the protein sequence coverage as identified by PEAKS Software. Reported sequences refer to the precursor proteins (i.e. they also contain the signal peptide). Signal peptide (when reported in the UniProtKB entry) is reported in a red rectangle. In legumin entries, a red arrow indicates the presumable C-terminal amino acid of the acidic (α) chain, as deduced by analogy with the homologous legumins of pea (Pisum sativum L.). In legumin se-quence entries A0A1S2XSB9, A0A3Q7XNW1, and A0A1S2XVG1 a blu rectangle holds the peptide, detected by MS data, carrying the C-term asparagine of the α-chain and the N-terminal glycine of the β-chain linked together. Peptides identified by LC-MS/MS are indicated by blue lines. Re-gions in the protein sequence that are covered by supporting peptides are displayed in bold font with a grey background. Confident modifications and amino acid mutations identified in sup-porting peptides are displayed as icons above the protein sequence. In detail, the oxidised me-thionine residues (M) (+15.99 Da) are indicated with a yellow "o"; the pyroglutamic residues (-17.03) are indicated with a blue "p"; carbamidomethyl-cysteines (+57.03) are reported with a red "c".

Figure S1

Vicilin Acc. No. A0A1S2YZ56 – Mass 81,9 kDa
(no information are reported for the peptide signal in the UniProtKB database)

Band N.7 (55 kDa)

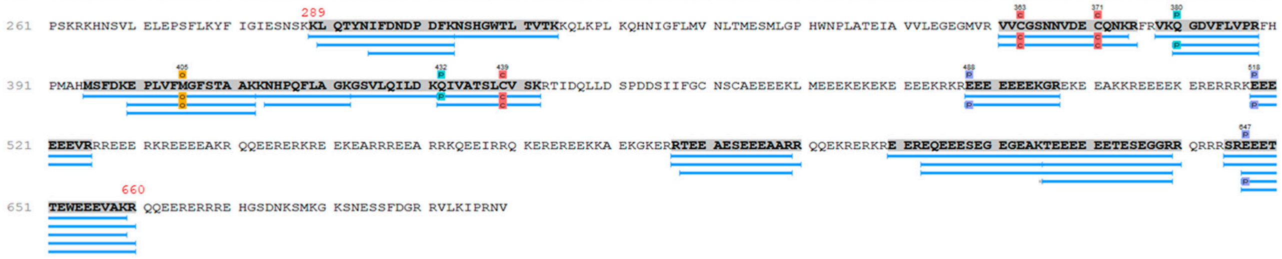

Band N.16 (24 kDa)

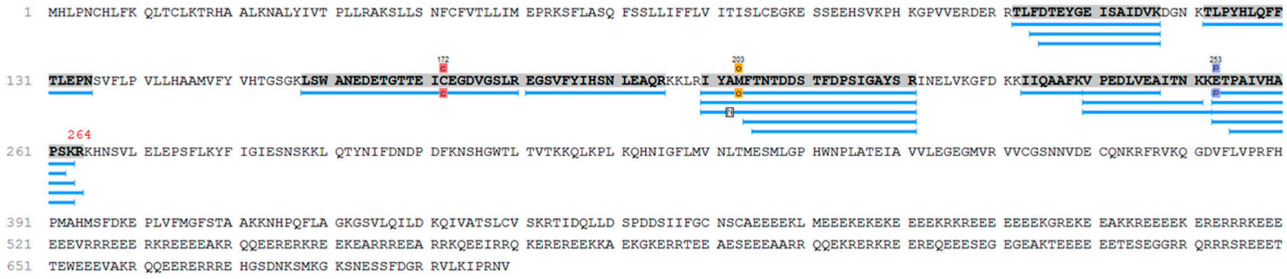

Figure S2

Vicilin Acc. No. A0A1S2XV08 – Mass 46,1 kDa (as mature protein)

Band N.10 (kDa 41)

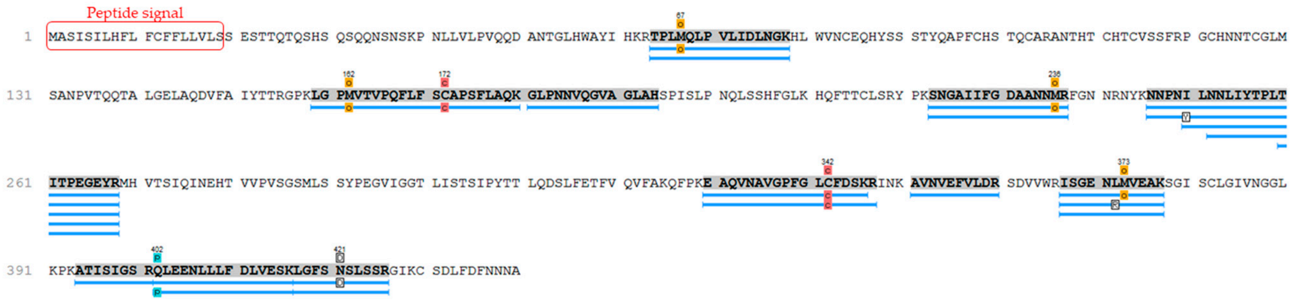

Band N.11 (kDa 38)

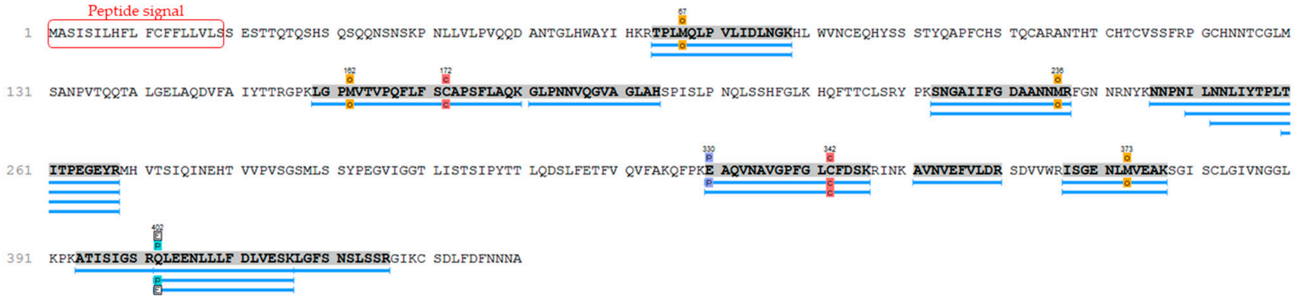

Figure S3

Vicilin Acc. No. A0A1S2Y087 - Mass 66,4 kDa (as mature protein)

Band N.2 (100 kDa) - (as minor component)

Peptide signal

```
  1 MATTTKTRFP LLLLLGIIFL ASVCVSYGIV QYEQGNPCLK KCMQRYNKVN EAFKRREEEE YEERSEHSVP GQRERGRQEG EKEEEHREQR RSRRESERER EEDEQKQRHP HHEREEEEDE EFRERRHRGD
131 TEETRFRRRK ERRQEQQEKK SESKEDEEES SSESQGRRNP FFFRSNKFQT IFENQHGHIR LLQNFDKRSN LFQNLQNYRF LEYKARPHTL LLPQHIDADF ILVVLSGKAI LTVLNPNDRN SFNLERGDTI
261 KLPAGTTAYL ANHDDEEDLR VVELAIPVNR PGKFQSFFPS SNQNQQSYFN GFSKSILEAS FNTKYETIER VLLEEQEPEQ SRGRRGSEES EEGDAIVKVS REQIEELSKH AKSSSRKSIS SESEPFNLRS
391 RNPIYSNKFG KFFEITPEKS PQLQDLNIFV SCVEINEGGL MLPHFNSRAI VVLFINEGKG HLELVGLRNE QQEQQQEEDE EQEEEERNNQ VQRFARLSP GDVFIIPAGH PVAVNASSDL NFVGFGINAE
521 NNQRNFLAGE DDNVISQIQN PVKELTFPGS AQEVNRLIKN QRQSYFANAQ PQQREEESQR RRGSLSSILG GFY
```

Band N.5 (65 kDa)

Peptide signal

```
  1 MATTTKTRFP LLLLLGIIFL ASVCVSYGIV QYEQGNPCLK KCMQRYNKVN EAFKRREEEE YEERSEHSVP GQRERGRQEG EKEEEHREQR RSRRESERER EEDEQKQRHP HHEREEEEDE EFRERRHRGD
131 TEETRFRRRK ERRQEQQEKK SESKEDEEES SSESQGRRNP FFFRSNKFQT IFENQHGHIR LLQNFDKRSN LFQNLQNYRF LEYKARPHTL LLPQHIDADF ILVVLSGKAI LTVLNPNDRN SFNLERGDTI
261 KLPAGTTAYL ANHDDEEDLR VVELAIPVNR PGKFQSFFPS SNQNQQSYFN GFSKSILEAS FNTKYETIER VLLEEQEPEQ SRGRRGSEES EEGDAIVKVS REQIEELSKH AKSSSRKSIS SESEPFNLRS
391 RNPIYSNKFG KFFEITPEKS PQLQDLNIFV SCVEINEGGL MLPHFNSRAI VVLFINEGKG HLELVGLRNE QQEQQQEEDE EQEEEERNNQ VQRFARLSP GDVFIIPAGH PVAVNASSDL NFVGFGINAE
521 NNQRNFLAGE DDNVISQIQN PVKELTFPGS AQEVNRLIKN QRQSYFANAQ PQQREEESQR RRGSLSSILG GFY
```

Band N.6 (60 kDa)

Peptide signal

```
  1 MATTTKTRFP LLLLLGIIFL ASVCVSYGIV QYEQGNPCLK KCMQRYNKVN EAFKRREEEE YEERSEHSVP GQRERGRQEG EKEEEHREQR RSRRESERER EEDEQKQRHP HHEREEEEDE EFRERRHRGD
131 TEETRFRRRK ERRQEQQEKK SESKEDEEES SSESQGRRNP FFFRSNKFQT IFENQHGHIR LLQNFDKRSN LFQNLQNYRF LEYKARPHTL LLPQHIDADF ILVVLSGKAI LTVLNPNDRN SFNLERGDTI
261 KLPAGTTAYL ANHDDEEDLR VVELAIPVNR PGKFQSFFPS SNQNQQSYFN GFSKSILEAS FNTKYETIER VLLEEQEPEQ SRGRRGSEES EEGDAIVKVS REQIEELSKH AKSSSRKSIS SESEPFNLRS
391 RNPIYSNKFG KFFEITPEKS PQLQDLNIFV SCVEINEGGL MLPHFNSRAI VVLFINEGKG HLELVGLRNE QQEQQQEEDE EQEEEERNNQ VQRFARLSP GDVFIIPAGH PVAVNASSDL NFVGFGINAE
521 NNQRNFLAGE DDNVISQIQN PVKELTFPGS AQEVNRLIKN QRQSYFANAQ PQQREEESQR RRGSLSSILG GFY
```

Band N.7 (55 kDa) - (as minor component)

Peptide signal

```
  1 MATTTKTRFP LLLLLGIIFL ASVCVSYGIV QYEQGNPCLK KCMQRYNKVN EAFKRREEEE YEERSEHSVP GQRERGRQEG EKEEEHREQR RSRRESERER EEDEQKQRHP HHEREEEEDE EFRERRHRGD
131 TEETRFRRRK ERRQEQQEKK SESKEDEEES SSESQGRRNP FFFRSNKFQT IFENQHGHIR LLQNFDKRSN LFQNLQNYRF LEYKARPHTL LLPQHIDADF ILVVLSGKAI LTVLNPNDRN SFNLERGDTI
261 KLPAGTTAYL ANHDDEEDLR VVELAIPVNR PGKFQSFFPS SNQNQQSYFN GFSKSILEAS FNTKYETIER VLLEEQEPEQ SRGRRGSEES EEGDAIVKVS REQIEELSKH AKSSSRKSIS SESEPFNLRS
391 RNPIYSNKFG KFFEITPEKS PQLQDLNIFV SCVEINEGGL MLPHFNSRAI VVLFINEGKG HLELVGLRNE QQEQQQEEDE EQEEEERNNQ VQRFARLSP GDVFIIPAGH PVAVNASSDL NFVGFGINAE
521 NNQRNFLAGE DDNVISQIQN PVKELTFPGS AQEVNRLIKN QRQSYFANAQ PQQREEESQR RRGSLSSILG GFY
```

Band N.8 (47 kDa)

Peptide signal

```
  1 MATTTKTRFP LLLLLGIIFL ASVCVSYGIV QYEQGNPCLK KCMQRYNKVN EAFKRREEEE YEERSEHSVP GQRERGRQEG EKEEEHREQR RSRRESERER EEDEQKQRHP HHEREEEEDE EFRERRHRGD
131 TEETRFRRRK ERRQEQQEKK SESKEDEEES SSESQGRRNP FFFRSNKFQT IFENQHGHIR LLQNFDKRSN LFQNLQNYRF LEYKARPHTL LLPQHIDADF ILVVLSGKAI LTVLNPNDRN SFNLERGDTI
261 KLPAGTTAYL ANHDDEEDLR VVELAIPVNR PGKFQSFFPS SNQNQQSYFN GFSKSILEAS FNTKYETIER VLLEEQEPEQ SRGRRGSEES EEGDAIVKVS REQIEELSKH AKSSSRKSIS SESEPFNLRS
391 RNPIYSNKFG KFFEITPEKS PQLQDLNIFV SCVEINEGGL MLPHFNSRAI VVLFINEGKG HLELVGLRNE QQEQQQEEDE EQEEEERNNQ VQRFARLSP GDVFIIPAGH PVAVNASSDL NFVGFGINAE
521 NNQRNFLAGE DDNVISQIQN PVKELTFPGS AQEVNRLIKN QRQSYFANAQ PQQREEESQR RRGSLSSILG GFY
```

Figure S4

Vicilin Acc. No. A0A1S2XYZ0 – Mass 61,9 kDa (as mature protein)

Band N.8 (47 kDa) - (as minor component)

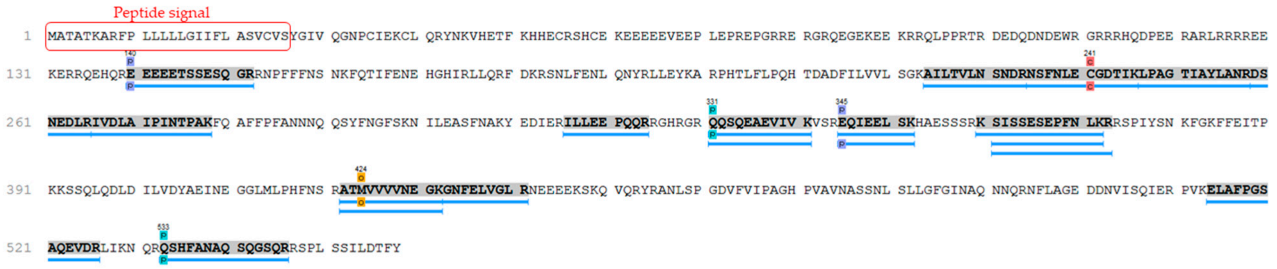

Band N.9 (44 kDa)

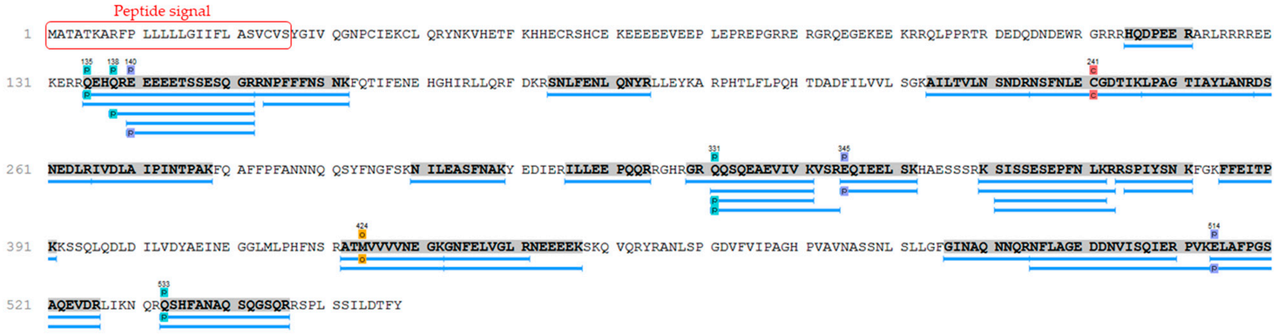

Figure S5

Vicilin Acc. No. A0A1S2XQR4 - Mass 49,4 kDa (as mature protein)

Band N.14 (32 kDa)

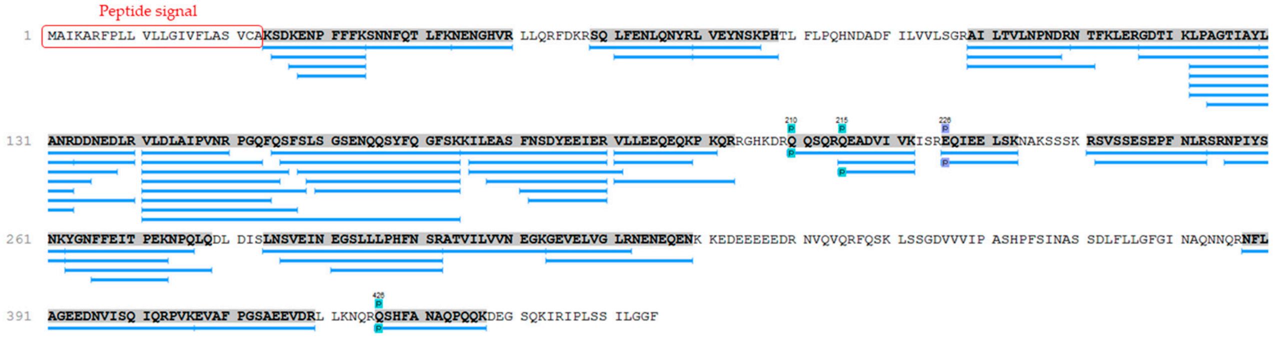

Figure S6

Vicilin Acc. No. A0A1S2XQ88 – Mass 48,6 kDa (as mature protein)

Band N.16 (24 kDa) - (as minor component)

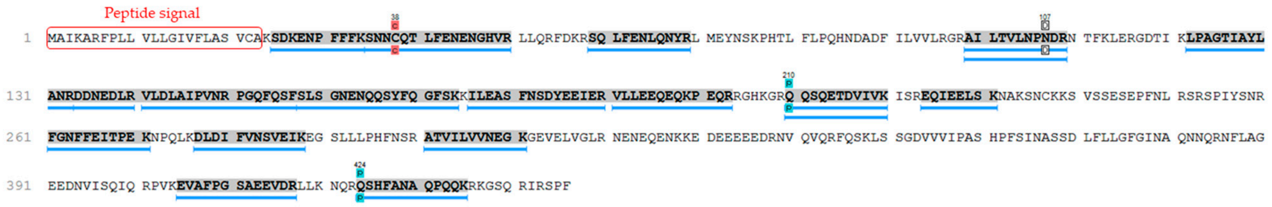

Band N.17 (23 kDa)

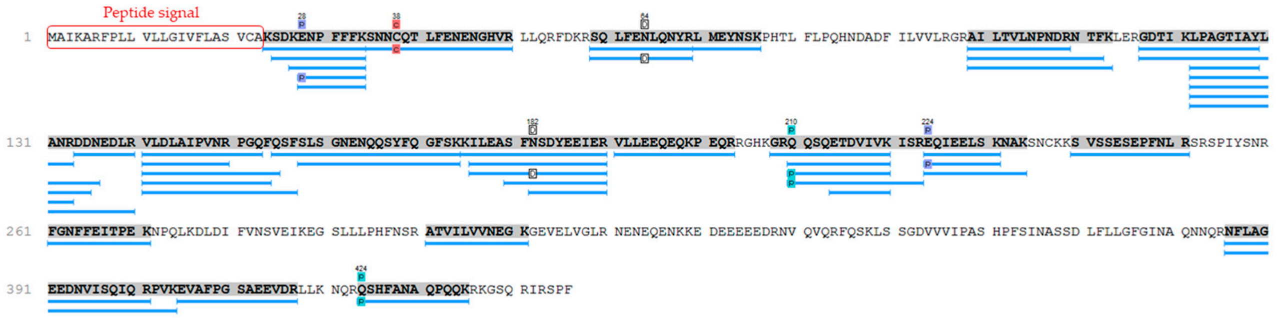

Figure S7

Legumin A-like A0A1S2XSB9 – Mass 57,1 kDa (as mature protein)
α-chain (trait Phe22-Asn333) – Mass 36,9 kDa
β-chain (trait Gly334- Ala518) – Mass 20,2 kDa

Band N.2 (100 kDa) – (as minor component)

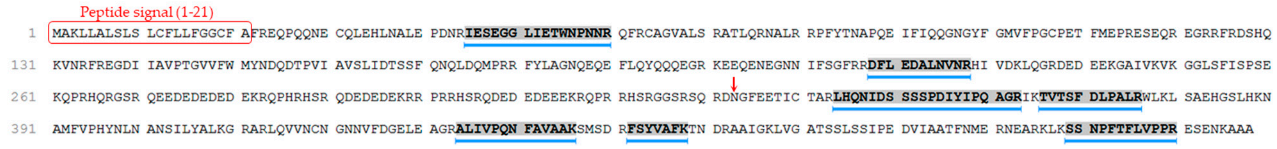

Band N.7 (55 kDa) - (as minor component)

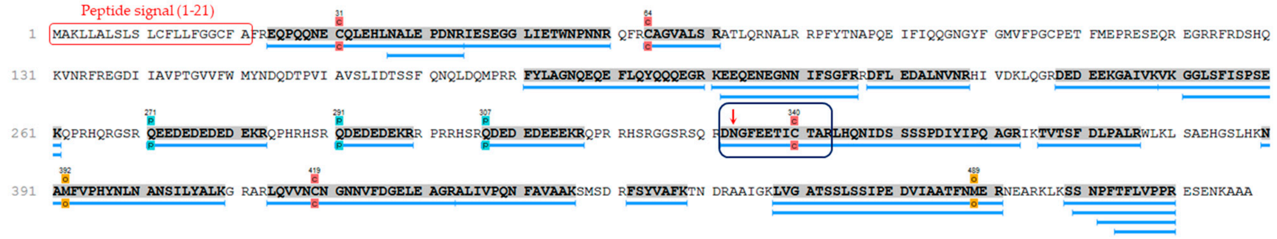

Band N.12 (36 kDa)

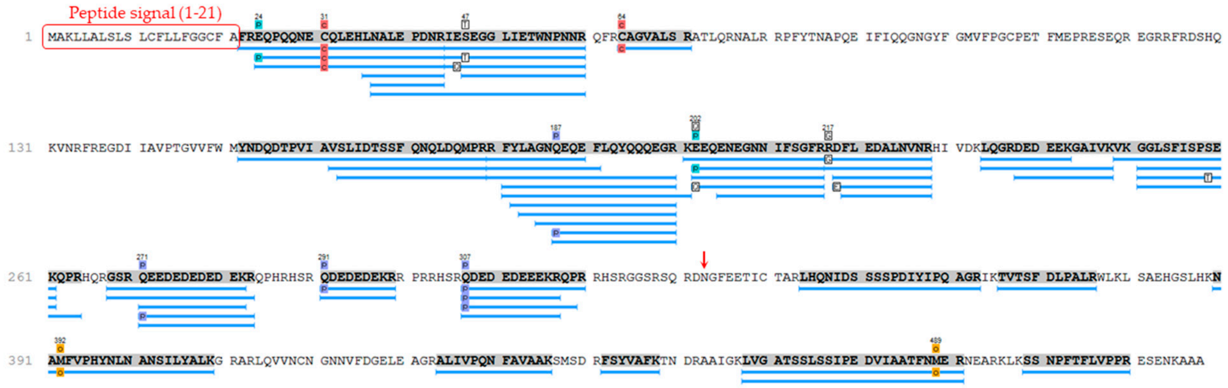

Band N.19 (20 kDa)

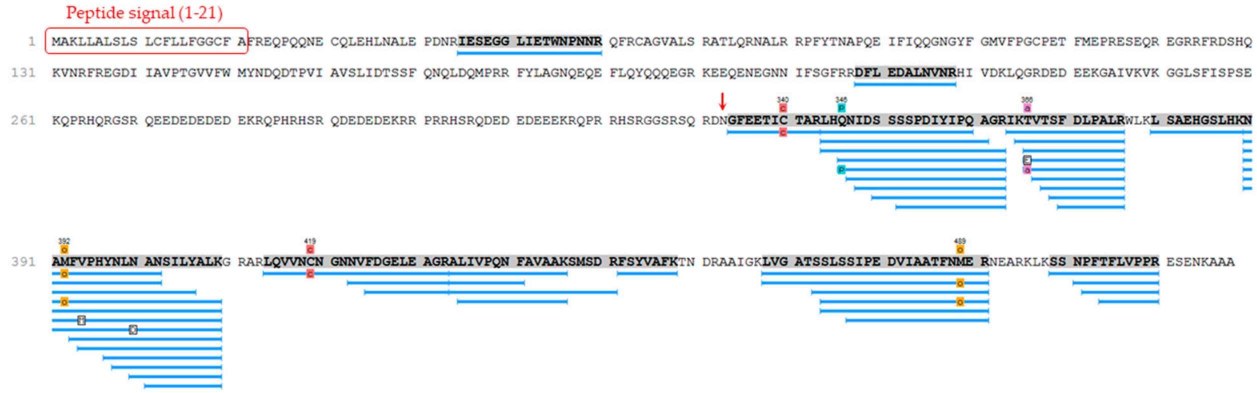

Figure S8

Legumin Acc. No. A0A3Q7XNW1 - Mass 54,0 kDa (as mature protein)
α-chain (trait Leu22-Asn314) – Mass 33,7 kDa
β-chain (trait Gly315- Ala500) – Mass 20,3 kDa

Band N.8 (47 kDa) - (as minor component)

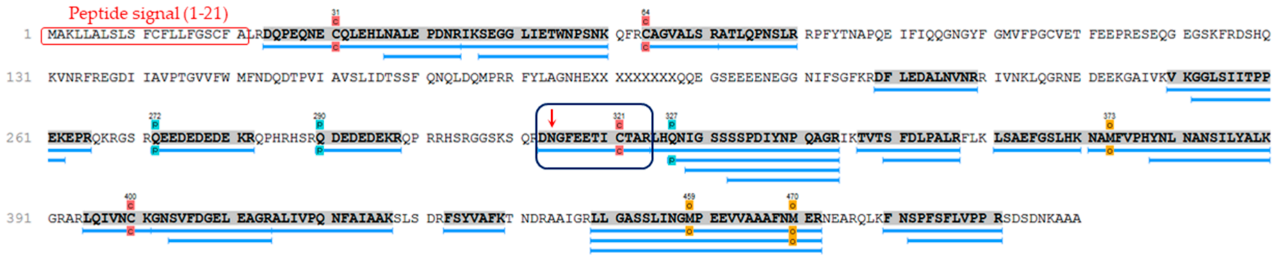

Band N.13 (34 kDa)

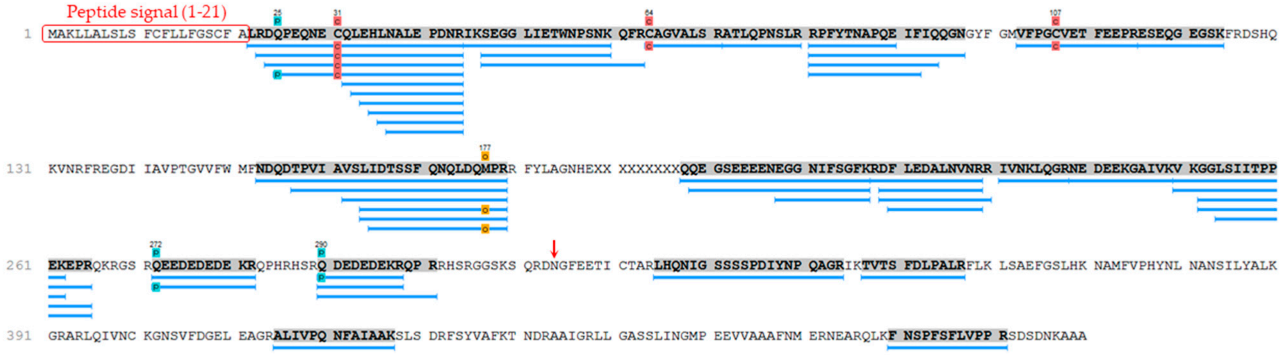

Band N.19 (20 kDa) - (as minor component)

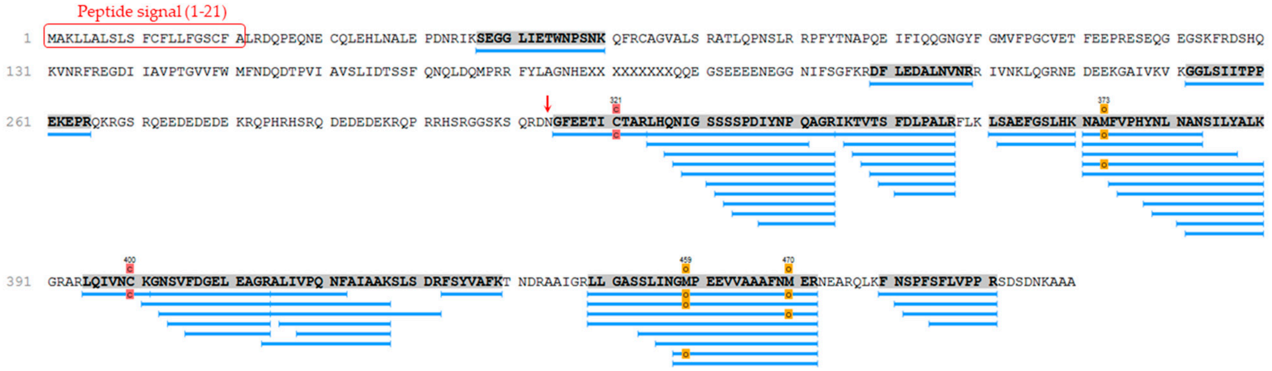

Figure S9

Legumin J Acc. No. A0A1S3E1N3 – Mass 59,6 kDa (as mature protein)

α-chain (trait Arg28-Asn363) – Mass 39,2 kDa

β-chain (trait Ser364- Leu551) – Mass 20,4 kDa

Band N.11 (38 kDa)

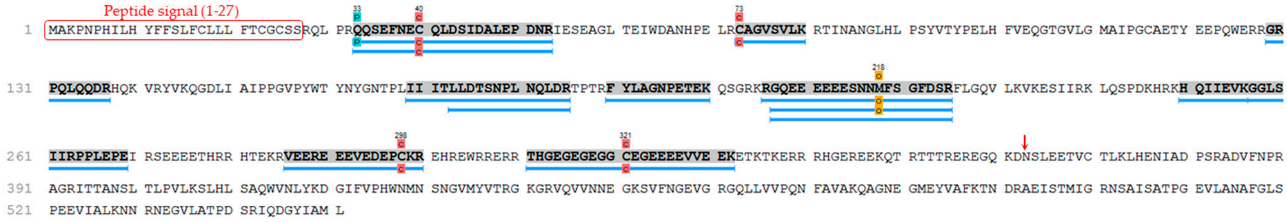

Band N.19 (20 kDa) – (as minor component)

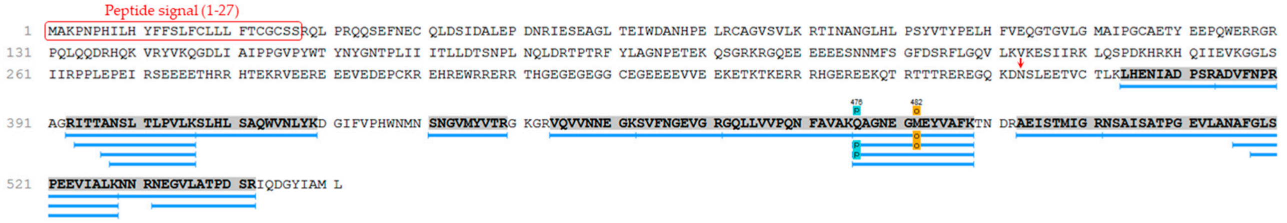

Figure S10

Legumin Acc. No. Q9SMJ4 – Mass 53,9 kDa (as mature protein)

α-chain (trait Leu22-Asn311) – Mass 33,3 kDa

β-chain (trait Ser312- Glu496) – Mass 20,6 kDa

Band N.13 (34 kDa)

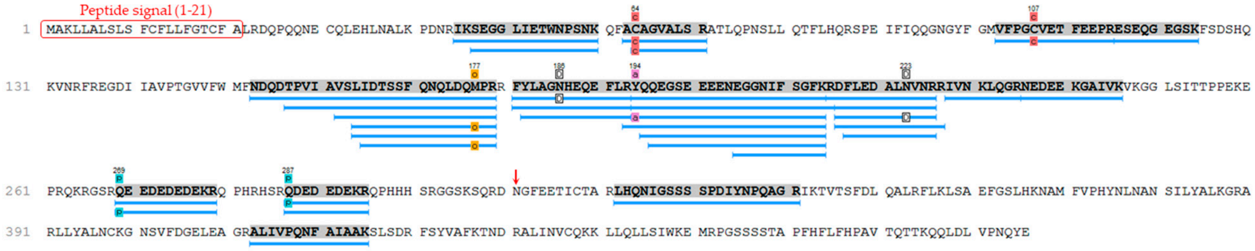

Figure S11

Legumin J-like Acc. No. A0A1S2XVG1 – Mass 57,7 kDa (as mature protein)
α-chain (trait Thr25-Asn352) – Mass 37,7 kDa
β-chain (trait Gly353- Ala532) – Mass 20,0 kDa

Band N.10 (41 kDa)

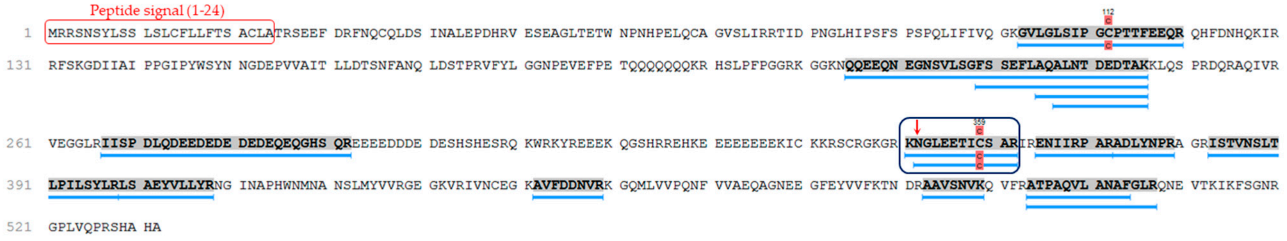

Band N.17 (23 kDa) – (as minor component)

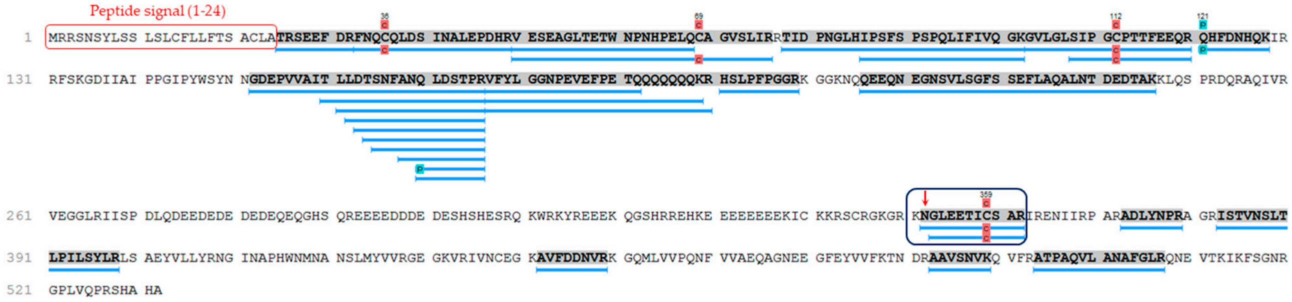

Band N.19 (20 kDa) – (as minor component)

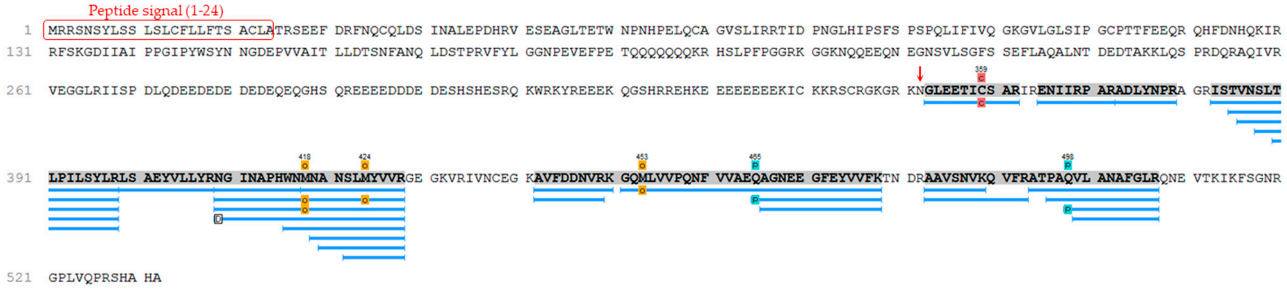

Band N.20 (20 kDa) – (as minor component)

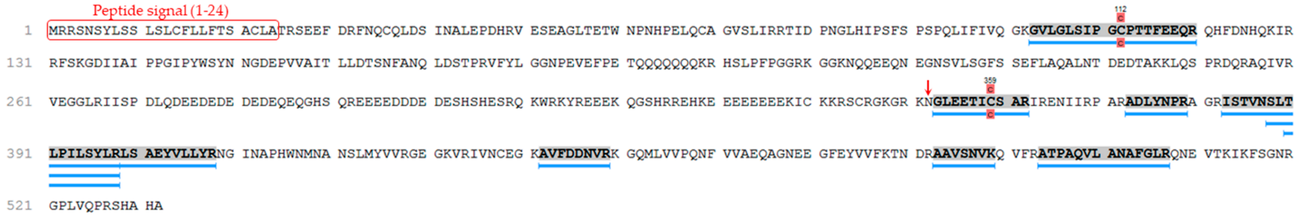

The vicilins with the Acc. Nos. Q304D4 and A0A1S2YKD9 were always identified as components in traces (relative abundance usually below 2%). Following their sequence coverage patterns.

Figure S12

Pro-Vicilin Acc. No. Q304D4– Mass 51,4 kDa

(no information are reported for the peptide signal in the UniProtKB database)

Band N.1 (150 kDa)

```
  1 MIVRFSLPDN ENDLKLTRSI NRDGEILIPK IFIIISVSQI SNGASREFDG ISSLKVEVFL SLGFNTVSIA LHLGLQDGSR HHCVVEERGC EVLSYFLQTV VLEVLKLLRT FVEPLEKTNV TVFVLEKSLK
131 VVRLKEKRIL LIGISHKLRP RKQFLSSTKS GNRALIAILM IEFLLSFRID DEIERVLLEE QEQKPKQRRG HKDRQQSQSQ SQQEADVIVK ISREQIEELS KNAKSSSKKS VSSESEPFNL RSRNPIYSNK
261 YGNFFEITPE KNPQLQDLDI SLNSVEINEG SLLLPHFNSR ATVILVVNEG KGEVELVGLR NENEQENKKE DEEEEEDRKV QVQRFQSRLS SGDVVVIPAT HPFSINASSD LFLLGFGINA QNNQRNFLAG
391 EEDNVISQIQ RPVKEVAFPG SAEEVDRLLK NQRQSHFANA QPQQKDEESQ KIRIPLSSIL GGF
```

Band N.2 (100 kDa)

```
  1 MIVRFSLPDN ENDLKLTRSI NRDGEILIPK IFIIISVSQI SNGASREFDG ISSLKVEVFL SLGFNTVSIA LHLGLQDGSR HHCVVEERGC EVLSYFLQTV VLEVLKLLRT FVEPLEKTNV TVFVLEKSLK
131 VVRLKEKRIL LIGISHKLRP RKQFLSSTKS GNRALIAILM IEFLLSFRID DEIERVLLEE QEQKPKQRRG HKDRQQSQSQ SQQEADVIVK ISREQIEELS KNAKSSSKKS VSSESEPFNL RSRNPIYSNK
261 YGNFFEITPE KNPQLQDLDI SLNSVEINEG SLLLPHFNSR ATVILVVNEG KGEVELVGLR NENEQENKKE DEEEEEDRKV QVQRFQSRLS SGDVVVIPAT HPFSINASSD LFLLGFGINA QNNQRNFLAG
391 EEDNVISQIQ RPVKEVAFPG SAEEVDRLLK NQRQSHFANA QPQQKDEESQ KIRIPLSSIL GGF
```

Band N.3 (90 kDa)

```
>tr|Q304D4|Q304D4_CICAR Provicilin OS=Cicer arietinum OX=3827 GN=LOC101515194 PE=2 SV=1
  1 MIVRFSLPDN ENDLKLTRSI NRDGEILIPK IFIIISVSQI SNGASREFDG ISSLKVEVFL SLGFNTVSIA LHLGLQDGSR HHCVVEERGC EVLSYFLQTV VLEVLKLLRT FVEPLEKTNV TVFVLEKSLK
131 VVRLKEKRIL LIGISHKLRP RKQFLSSTKS GNRALIAILM IEFLLSFRID DEIERVLLEE QEQKPKQRRG HKDRQQSQSQ SQQEADVIVK ISREQIEELS KNAKSSSKKS VSSESEPFNL RSRNPIYSNK
261 YGNFFEITPE KNPQLQDLDI SLNSVEINEG SLLLPHFNSR ATVILVVNEG KGEVELVGLR NENEQENKKE DEEEEEDRKV QVQRFQSRLS SGDVVVIPAT HPFSINASSD LFLLGFGINA QNNQRNFLAG
391 EEDNVISQIQ RPVKEVAFPG SAEEVDRLLK NQRQSHFANA QPQQKDEESQ KIRIPLSSIL GGF
```

Band N.4 (70 kDa)

```
  1 MIVRFSLPDN ENDLKLTRSI NRDGEILIPK IFIIISVSQI SNGASREFDG ISSLKVEVFL SLGFNTVSIA LHLGLQDGSR HHCVVEERGC EVLSYFLQTV VLEVLKLLRT FVEPLEKTNV TVFVLEKSLK
131 VVRLKEKRIL LIGISHKLRP RKQFLSSTKS GNRALIAILM IEFLLSFRID DEIERVLLEE QEQKPKQRRG HKDRQQSQSQ SQQEADVIVK ISREQIEELS KNAKSSSKKS VSSESEPFNL RSRNPIYSNK
261 YGNFFEITPE KNPQLQDLDI SLNSVEINEG SLLLPHFNSR ATVILVVNEG KGEVELVGLR NENEQENKKE DEEEEEDRKV QVQRFQSRLS SGDVVVIPAT HPFSINASSD LFLLGFGINA QNNQRNFLAG
391 EEDNVISQIQ RPVKEVAFPG SAEEVDRLLK NQRQSHFANA QPQQKDEESQ KIRIPLSSIL GGF
```

Band N.5 (65 kDa)

```
  1 MIVRFSLPDN ENDLKLTRSI NRDGEILIPK IFIIISVSQI SNGASREFDG ISSLKVEVFL SLGFNTVSIA LHLGLQDGSR HHCVVEERGC EVLSYFLQTV VLEVLKLLRT FVEPLEKTNV TVFVLEKSLK
131 VVRLKEKRIL LIGISHKLRP RKQFLSSTKS GNRALIAILM IEFLLSFRID DEIERVLLEE QEQKPKQRRG HKDRQQSQSQ SQQEADVIVK ISREQIEELS KNAKSSSKKS VSSESEPFNL RSRNPIYSNK
261 YGNFFEITPE KNPQLQDLDI SLNSVEINEG SLLLPHFNSR ATVILVVNEG KGEVELVGLR NENEQENKKE DEEEEEDRKV QVQRFQSRLS SGDVVVIPAT HPFSINASSD LFLLGFGINA QNNQRNFLAG
391 EEDNVISQIQ RPVKEVAFPG SAEEVDRLLK NQRQSHFANA QPQQKDEESQ KIRIPLSSIL GGF
```

Band N.6 (60 kDa)

```
  1 MIVRFSLPDN ENDLKLTRSI NRDGEILIPK IFIIISVSQI SNGASREFDG ISSLKVEVFL SLGFNTVSIA LHLGLQDGSR HHCVVEERGC EVLSYFLQTV VLEVLKLLRT FVEPLEKTNV TVFVLEKSLK
131 VVRLKEKRIL LIGISHKLRP RKQFLSSTKS GNRALIAILM IEFLLSFRID DEIERVLLEE QEQKPKQRRG HKDRQQSQSQ SQQEADVIVK ISREQIEELS KNAKSSSKKS VSSESEPFNL RSRNPIYSNK
261 YGNFFEITPE KNPQLQDLDI SLNSVEINEG SLLLPHFNSR ATVILVVNEG KGEVELVGLR NENEQENKKE DEEEEEDRKV QVQRFQSRLS SGDVVVIPAT HPFSINASSD LFLLGFGINA QNNQRNFLAG
391 EEDNVISQIQ RPVKEVAFPG SAEEVDRLLK NQRQSHFANA QPQQKDEESQ KIRIPLSSIL GGF
```

Band N.8 (47 kDa)

1 MIVRFSLPDN ENDLKLTRSI NRDGEILIPK IFIIISVSQI SNGASREFDG ISSLKVEVFL SLGFNTVSIA LHLGLQDGSR HHCVVEERGC EVLSYFLQTV VLEVLKLLRT FVEPLEKTNV TVFVLEKSLK
131 VVRLKEKRIL LIGISHKLRP RKQFLSSTKS GNRALIAILM IEFLLSFRID DEIERVLLEE QEQKPKQRRG HKDRQQSQSQ SQQEADVIVK ISREQIEELS KNAKSSSKKS VSSESEPFNL RSRNPIYSNK
261 YGNFFEITPE KNPQLQDLDI SLNSVEINEG SLLLPHFNSR ATVILVVNEG KGEVELVGLR NENEQENKKE DEEEEEDRKV QVQRFQSRLS SGDVVVIPAT HPFSINASSD LFLLGFGINA QNNQRNFLAG
391 EEDNVISQIQ RPVKEVAFPG SAEEVDRLLK NQRQSHFANA QPQQKDEESQ KIRIPLSSIL GGF

Band N.9 (44 kDa)

1 MIVRFSLPDN ENDLKLTRSI NRDGEILIPK IFIIISVSQI SNGASREFDG ISSLKVEVFL SLGFNTVSIA LHLGLQDGSR HHCVVEERGC EVLSYFLQTV VLEVLKLLRT FVEPLEKTNV TVFVLEKSLK
131 VVRLKEKRIL LIGISHKLRP RKQFLSSTKS GNRALIAILM IEFLLSFRID DEIERVLLEE QEQKPKQRRG HKDRQQSQSQ SQQEADVIVK ISREQIEELS KNAKSSSKKS VSSESEPFNL RSRNPIYSNK
261 YGNFFEITPE KNPQLQDLDI SLNSVEINEG SLLLPHFNSR ATVILVVNEG KGEVELVGLR NENEQENKKE DEEEEEDRKV QVQRFQSRLS SGDVVVIPAT HPFSINASSD LFLLGFGINA QNNQRNFLAG
391 EEDNVISQIQ RPVKEVAFPG SAEEVDRLLK NQRQSHFANA QPQQKDEESQ KIRIPLSSIL GGF

Band N.10 (41 kDa)

1 MIVRFSLPDN ENDLKLTRSI NRDGEILIPK IFIIISVSQI SNGASREFDG ISSLKVEVFL SLGFNTVSIA LHLGLQDGSR HHCVVEERGC EVLSYFLQTV VLEVLKLLRT FVEPLEKTNV TVFVLEKSLK
131 VVRLKEKRIL LIGISHKLRP RKQFLSSTKS GNRALIAILM IEFLLSFRID DEIERVLLEE QEQKPKQRRG HKDRQQSQSQ SQQEADVIVK ISREQIEELS KNAKSSSKKS VSSESEPFNL RSRNPIYSNK
261 YGNFFEITPE KNPQLQDLDI SLNSVEINEG SLLLPHFNSR ATVILVVNEG KGEVELVGLR NENEQENKKE DEEEEEDRKV QVQRFQSRLS SGDVVVIPAT HPFSINASSD LFLLGFGINA QNNQRNFLAG
391 EEDNVISQIQ RPVKEVAFPG SAEEVDRLLK NQRQSHFANA QPQQKDEESQ KIRIPLSSIL GGF

Band N.11 (38 kDa)

1 MIVRFSLPDN ENDLKLTRSI NRDGEILIPK IFIIISVSQI SNGASREFDG ISSLKVEVFL SLGFNTVSIA LHLGLQDGSR HHCVVEERGC EVLSYFLQTV VLEVLKLLRT FVEPLEKTNV TVFVLEKSLK
131 VVRLKEKRIL LIGISHKLRP RKQFLSSTKS GNRALIAILM IEFLLSFRID DEIERVLLEE QEQKPKQRRG HKDRQQSQSQ SQQEADVIVK ISREQIEELS KNAKSSSKKS VSSESEPFNL RSRNPIYSNK
261 YGNFFEITPE KNPQLQDLDI SLNSVEINEG SLLLPHFNSR ATVILVVNEG KGEVELVGLR NENEQENKKE DEEEEEDRKV QVQRFQSRLS SGDVVVIPAT HPFSINASSD LFLLGFGINA QNNQRNFLAG
391 EEDNVISQIQ RPVKEVAFPG SAEEVDRLLK NQRQSHFANA QPQQKDEESQ KIRIPLSSIL GGF

Band N.12 (36 kDa)

1 MIVRFSLPDN ENDLKLTRSI NRDGEILIPK IFIIISVSQI SNGASREFDG ISSLKVEVFL SLGFNTVSIA LHLGLQDGSR HHCVVEERGC EVLSYFLQTV VLEVLKLLRT FVEPLEKTNV TVFVLEKSLK
131 VVRLKEKRIL LIGISHKLRP RKQFLSSTKS GNRALIAILM IEFLLSFRID DEIERVLLEE QEQKPKQRRG HKDRQQSQSQ SQQEADVIVK ISREQIEELS KNAKSSSKKS VSSESEPFNL RSRNPIYSNK
261 YGNFFEITPE KNPQLQDLDI SLNSVEINEG SLLLPHFNSR ATVILVVNEG KGEVELVGLR NENEQENKKE DEEEEEDRKV QVQRFQSRLS SGDVVVIPAT HPFSINASSD LFLLGFGINA QNNQRNFLAG
391 EEDNVISQIQ RPVKEVAFPG SAEEVDRLLK NQRQSHFANA QPQQKDEESQ KIRIPLSSIL GGF

Band N.13 (34 kDa)

1 MIVRFSLPDN ENDLKLTRSI NRDGEILIPK IFIIISVSQI SNGASREFDG ISSLKVEVFL SLGFNTVSIA LHLGLQDGSR HHCVVEERGC EVLSYFLQTV VLEVLKLLRT FVEPLEKTNV TVFVLEKSLK
131 VVRLKEKRIL LIGISHKLRP RKQFLSSTKS GNRALIAILM IEFLLSFRID DEIERVLLEE QEQKPKQRRG HKDRQQSQSQ SQQEADVIVK ISREQIEELS KNAKSSSKKS VSSESEPFNL RSRNPIYSNK
261 YGNFFEITPE KNPQLQDLDI SLNSVEINEG SLLLPHFNSR ATVILVVNEG KGEVELVGLR NENEQENKKE DEEEEEDRKV QVQRFQSRLS SGDVVVIPAT HPFSINASSD LFLLGFGINA QNNQRNFLAG
391 EEDNVISQIQ RPVKEVAFPG SAEEVDRLLK NQRQSHFANA QPQQKDEESQ KIRIPLSSIL GGF

Band N.14 (32 kDa)

1 MIVRFSLPDN ENDLKLTRSI NRDGEILIPK IFIIISVSQI SNGASREFDG ISSLKVEVFL SLGFNTVSIA LHLGLQDGSR HHCVVEERGC EVLSYFLQTV VLEVLKLLRT FVEPLEKTNV TVFVLEKSLK
131 VVRLKEKRIL LIGISHKLRP RKQFLSSTKS GNRALIAILM IEFLLSFRID DEIERVLLEE QEQKPKQRRG HKDRQQSQSQ SQQEADVIVK ISREQIEELS KNAKSSSKKS VSSESEPFNL RSRNPIYSNK
261 YGNFFEITPE KNPQLQDLDI SLNSVEINEG SLLLPHFNSR ATVILVVNEG KGEVELVGLR NENEQENKKE DEEEEEDRKV QVQRFQSRLS SGDVVVIPAT HPFSINASSD LFLLGFGINA QNNQRNFLAG
391 EEDNVISQIQ RPVKEVAFPG SAEEVDRLLK NQRQSHFANA QPQQKDEESQ KIRIPLSSIL GGF

Band N.15 (30 kDa)

1 MIVRFSLPDN ENDLKLTRSI NRDGEILIPK IFIIISVSQI SNGASREFDG ISSLKVEVFL SLGFNTVSIA LHLGLQDGSR HHCVVEERGC EVLSYFLQTV VLEVLKLLRT FVEPLEKTNV TVFVLEKSLK

131 VVRLKEKRIL LIGISHKLRP RKQFLSSTKS GNRALIAILM IEFLLSFRID DEIERVLLEE QEQKPKQRRG HKDRQQSQSQ SQQEADVIVK ISREQIEELS KNAKSSSKKS VSSESEPFNL RSRNPIYSNK

261 YGNFFEITPE KNPQLQDLDI SLNSVEINEG SLLLPHFNSR ATVILVVNEG KGEVELVGLR NENEQENKKE DEEEEEDRKV QVQRFQSRLS SGDVVVIPAT HPFSINASSD LFLLGFGINA QNNQRNFLAG

391 EEDNVISQIQ RPVKEVAFPG SAEEVDRLLK NQRQSHFANA QPQQKDEESQ KIRIPLSSIL GGF

Band N.16 (24 kDa)

1 MIVRFSLPDN ENDLKLTRSI NRDGEILIPK IFIIISVSQI SNGASREFDG ISSLKVEVFL SLGFNTVSIA LHLGLQDGSR HHCVVEERGC EVLSYFLQTV VLEVLKLLRT FVEPLEKTNV TVFVLEKSLK

131 VVRLKEKRIL LIGISHKLRP RKQFLSSTKS GNRALIAILM IEFLLSFRID DEIERVLLEE QEQKPKQRRG HKDRQQSQSQ SQQEADVIVK ISREQIEELS KNAKSSSKKS VSSESEPFNL RSRNPIYSNK

261 YGNFFEITPE KNPQLQDLDI SLNSVEINEG SLLLPHFNSR ATVILVVNEG KGEVELVGLR NENEQENKKE DEEEEEDRKV QVQRFQSRLS SGDVVVIPAT HPFSINASSD LFLLGFGINA QNNQRNFLAG

391 EEDNVISQIQ RPVKEVAFPG SAEEVDRLLK NQRQSHFANA QPQQKDEESQ KIRIPLSSIL GGF

Band N.17 (23 kDa)

1 MIVRFSLPDN ENDLKLTRSI NRDGEILIPK IFIIISVSQI SNGASREFDG ISSLKVEVFL SLGFNTVSIA LHLGLQDGSR HHCVVEERGC EVLSYFLQTV VLEVLKLLRT FVEPLEKTNV TVFVLEKSLK

131 VVRLKEKRIL LIGISHKLRP RKQFLSSTKS GNRALIAILM IEFLLSFRID DEIERVLLEE QEQKPKQRRG HKDRQQSQSQ SQQEADVIVK ISREQIEELS KNAKSSSKKS VSSESEPFNL RSRNPIYSNK

261 YGNFFEITPE KNPQLQDLDI SLNSVEINEG SLLLPHFNSR ATVILVVNEG KGEVELVGLR NENEQENKKE DEEEEEDRKV QVQRFQSRLS SGDVVVIPAT HPFSINASSD LFLLGFGINA QNNQRNFLAG

391 EEDNVISQIQ RPVKEVAFPG SAEEVDRLLK NQRQSHFANA QPQQKDEESQ KIRIPLSSIL GGF

Band N.18 (22 kDa)

1 MIVRFSLPDN ENDLKLTRSI NRDGEILIPK IFIIISVSQI SNGASREFDG ISSLKVEVFL SLGFNTVSIA LHLGLQDGSR HHCVVEERGC EVLSYFLQTV VLEVLKLLRT FVEPLEKTNV TVFVLEKSLK

131 VVRLKEKRIL LIGISHKLRP RKQFLSSTKS GNRALIAILM IEFLLSFRID DEIERVLLEE QEQKPKQRRG HKDRQQSQSQ SQQEADVIVK ISREQIEELS KNAKSSSKKS VSSESEPFNL RSRNPIYSNK

261 YGNFFEITPE KNPQLQDLDI SLNSVEINEG SLLLPHFNSR ATVILVVNEG KGEVELVGLR NENEQENKKE DEEEEEDRKV QVQRFQSRLS SGDVVVIPAT HPFSINASSD LFLLGFGINA QNNQRNFLAG

391 EEDNVISQIQ RPVKEVAFPG SAEEVDRLLK NQRQSHFANA QPQQKDEESQ KIRIPLSSIL GGF

Band N.19 (20 kDa)

1 MIVRFSLPDN ENDLKLTRSI NRDGEILIPK IFIIISVSQI SNGASREFDG ISSLKVEVFL SLGFNTVSIA LHLGLQDGSR HHCVVEERGC EVLSYFLQTV VLEVLKLLRT FVEPLEKTNV TVFVLEKSLK

131 VVRLKEKRIL LIGISHKLRP RKQFLSSTKS GNRALIAILM IEFLLSFRID DEIERVLLEE QEQKPKQRRG HKDRQQSQSQ SQQEADVIVK ISREQIEELS KNAKSSSKKS VSSESEPFNL RSRNPIYSNK

261 YGNFFEITPE KNPQLQDLDI SLNSVEINEG SLLLPHFNSR ATVILVVNEG KGEVELVGLR NENEQENKKE DEEEEEDRKV QVQRFQSRLS SGDVVVIPAT HPFSINASSD LFLLGFGINA QNNQRNFLAG

391 EEDNVISQIQ RPVKEVAFPG SAEEVDRLLK NQRQSHFANA QPQQKDEESQ KIRIPLSSIL GGF

Band N.20 (20 kDa)

1 MIVRFSLPDN ENDLKLTRSI NRDGEILIPK IFIIISVSQI SNGASREFDG ISSLKVEVFL SLGFNTVSIA LHLGLQDGSR HHCVVEERGC EVLSYFLQTV VLEVLKLLRT FVEPLEKTNV TVFVLEKSLK

131 VVRLKEKRIL LIGISHKLRP RKQFLSSTKS GNRALIAILM IEFLLSFRID DEIERVLLEE QEQKPKQRRG HKDRQQSQSQ SQQEADVIVK ISREQIEELS KNAKSSSKKS VSSESEPFNL RSRNPIYSNK

261 YGNFFEITPE KNPQLQDLDI SLNSVEINEG SLLLPHFNSR ATVILVVNEG KGEVELVGLR NENEQENKKE DEEEEEDRKV QVQRFQSRLS SGDVVVIPAT HPFSINASSD LFLLGFGINA QNNQRNFLAG

391 EEDNVISQIQ RPVKEVAFPG SAEEVDRLLK NQRQSHFANA QPQQKDEESQ KIRIPLSSIL GGF

Figure S13

Vicilin Acc. No. A0A1S2YKD9 – Mass 50,9 kDa (as mature protein)

Band N.16 (24 kDa)

Peptide signal

1 MTKNITTLLL LLLVGVTITL CEDEEREKGS SSSNKLFLMQ DSKSVVKTDA GEMRLFQNKD TEFLDRNMHI GLINMEPRSL FIPQYLDSNF IIFVRRGDAK LGFIYGDELE ERRLKTGDIY VIPAGSVFYL
131 VNIGEGQRLH LICSIDPSTS LGDTFQPFYI GGGDNSQSVL AGFEPVILET AFNESREKLE RIFSKKVDGP IVYIDDSHAP SLWTKFLQLK KEEKVQHLNK MVQGQEENNE EKKQTSWSWR KLFQSVLGEE
261 NKNIENKDR**G DSPDSYNLYD R**KHDFKNGYG WSSALDGDDY SLLKIPDIGV FHVNLTAGSM MVPHVNPR**AT EYGIVLR**GSG RIQILFPNGS NAMDTEIK**VG DIFYVPR**YFP FCQIASR**NGA LEFFGFTTSS**
391 **KKSNPQFLVG AASLLKTIMG PELAAGFGVR** EDIMR**DVVGA QHDAVIVSST WAAPGTGGVR** EEDAYVQSKG IIGIFDDILM DDFE

Band N.17 (23 kDa)

Peptide signal

1 MTKNITTLLL LLLVGVTITL CEDEEREKGS SSSNKLFLMQ DSKSVVKTDA GEMRLFQNKD TEFLDRNMHI GLINMEPRSL FIPQYLDSNF IIFVRRGDAK LGFIYGDELE ERRLKTGDIY VIPAGSVFYL
131 VNIGEGQRLH LICSIDPSTS LGDTFQPFYI GGGDNSQSVL AGFEPVILET AFNESREKLE RIFSKKVDGP IVYIDDSHAP SLWTKFLQLK KEEKVQHLNK MVQGQEENNE EKKQTSWSWR KLFQSVLGEE
261 NKNIENKDR**G DSPDSYNLYD R**KHDFKNGYG WSSALDGDDY SLLKIPDIGV FHVNLTAGSM MVPHVNPR**AT EYGIVLR**GSG RIQILFPNGS NAMDTEIK**VG DIFYVPRYFP FCQIASRNGA LEFFGFTTSS**
391 **KKSNPQFLVG AASLLKTIMG PELAAGFGVR** EDIMR**DVVGA QHDAVIVSST WAAPGTGG**VR EEDAYVQSKG IIGIFDDILM DDFE

Band N.18 (22 kDa)

Peptide signal

1 MTKNITTLLL LLLVGVTITL CEDEEREKGS SSSNKLFLMQ DSKSVVKTDA GEMRLFQNKD TEFLDRNMHI GLINMEPRSL FIPQYLDSNF IIFVRRGDAK LGFIYGDELE ERRLKTGDIY VIPAGSVFYL
131 VNIGEGQRLH LICSIDPSTS LGDTFQPFYI GGGDNSQSVL AGFEPVILET AFNESREKLE RIFSKKVDGP IVYIDDSHAP SLWTKFLQLK KEEKVQHLNK MVQGQEENNE EKKQTSWSWR KLFQSVLGEE
261 NKNIENKDRG DSPDSYNLYD RKHDFKNGYG WSSALDGDDY SLLKIPDIGV FHVNLTAGSM MVPHVNPR**AT EYGIVLR**GSG RIQILFPNGS NAMDTEIKVG DIFYVPRYFP FCQIASRNGA LEFFGFTTSS
391 K**KSNPQFLVG AASLLK**TIMG PELAAGFGVR EDIMRDVVGA QHDAVIVSST WAAPGTGGVR EEDAYVQSKG IIGIFDDILM DDFE

Band N.19 (20 kDa)

Peptide signal

1 MTKNITTLLL LLLVGVTITL CEDEEREKGS SSSNK**LFLMQ DSK**SVVKTDA GEMRLFQNKD TEFLDR**NMHI GLINMEPR**SL FIPQYLDSNF IIFVRRGDAK **LGFIYGDELE ER**RLKTGDIY VIPAGSVFYL
131 VNIGEGQRLH LICSIDPSTS LGDTFQPFYI GGGDNSQSVL AGFEPVILET AFNESREKLE RIFSKKVDGP IVYIDDSHAP SLWTKFLQLK KEEKVQHLNK MVQGQEENNE EKKQTSWSWR KLFQSVLGEE
261 NKNIENKDRG DSPDSYNLYD RKHDFKNGYG WSSALDGDDY SLLKIPDIGV FHVNLTAGSM MVPHVNPRAT EYGIVLRGSG RIQILFPNGS NAMDTEIKVG DIFYVPRYFP FCQIASRNGA LEFFGFTTSS
391 KKSNPQFLVG AASLLKTIMG PELAAGFGVR EDIMRDVVGA QHDAVIVSST WAAPGTGGVR EEDAYVQSKG IIGIFDDILM DDFE
